# A Structural Update of Neutralizing Epitopes on the HIV Envelope, a Moving Target

**DOI:** 10.3390/v13091774

**Published:** 2021-09-05

**Authors:** Emma Parker Miller, Maxwell T. Finkelstein, Molly C. Erdman, Paul C. Seth, Daniela Fera

**Affiliations:** Department of Chemistry and Biochemistry, Swarthmore College, Swarthmore, PA 19081, USA; emiller6@swarthmore.edu (E.P.M.); mfinkel1@swarthmore.edu (M.T.F.); merdman1@swarthmore.edu (M.C.E.); pseth1@swarthmore.edu (P.C.S.)

**Keywords:** HIV-1, envelope, spike, neutralizing antibodies, epitope, neutralization, structure

## Abstract

Antibodies that can neutralize diverse HIV-1 strains develop in ~10–20% of HIV-1 infected individuals, and their elicitation is a goal of vaccine design. Such antibodies can also serve as therapeutics for those who have already been infected with the virus. Structural characterizations of broadly reactive antibodies in complex with the HIV-1 spike indicate that there are a limited number of sites of vulnerability on the spike. Analysis of their structures can help reveal commonalities that would be useful in vaccine design and provide insights on combinations of antibodies that can be used to minimize the incidence of viral resistance mutations. In this review, we give an update on recent structures determined of the spike in complex with broadly neutralizing antibodies in the context of all epitopes on the HIV-1 spike identified to date.

## 1. Introduction

HIV-1 is a rapidly evolving pathogen, which has necessitated the use of antiretroviral therapies that use cocktails of drugs, to treat infected individuals while minimizing the development of resistance. Antibodies, which have longer half-lives in vivo, are increasingly being explored for their potential use both therapeutically and prophylactically [1,2,3,4,5,6]. Despite the high levels of sequence variation in the HIV-1 spike protein, called the envelope, or “Env”, neutralizing antibodies (nAbs) against semi-conserved regions have been identified. Non-neutralizing HIV-1 antibodies have also been identified and contribute to the in vivo protective immune response, but they are rarely, if ever, structurally characterized [7,8]. Since the majority of available structural information on antibody-based immune responses to HIV is based on neutralizing antibodies, these are the focus of this review. Of particular interest are broadly neutralizing antibodies (bnAbs), which are effective at targeting a wide range of viral strains. BnAbs are the focus of HIV-1 vaccine design programs and have utility in the development of therapeutics.

The first bnAbs were identified in the early 1990s, including b12 against the CD4 binding site (CD4bs), using a phage display library from an asymptomatic infected individual [9,10]. Since then, advances in single B-cell sorting technologies and next-generation sequencing have made possible more efficient identification of bnAbs from the sera of infected individuals [11]. To date, bnAbs have been identified against six major epitopes on HIV-1 Env, essentially covering all the accessible sites. Structural features of many of these sites have been reviewed elsewhere [12,13,14]. In this review, we focus on bnAbs that have been more recently structurally characterized in complex with Env.

Understanding how these bnAbs interact with their respective epitopes contributes to immunogen design for eliciting bnAbs against HIV-1 by vaccination, and aids the formulation of effective antibody combinations for therapeutic use. The use or elicitation of antibodies with different epitopes or hotspots reduces the chance of viral escape [11,15].

## 2. HIV-1 Env Structure and Function

**Figure 1:**
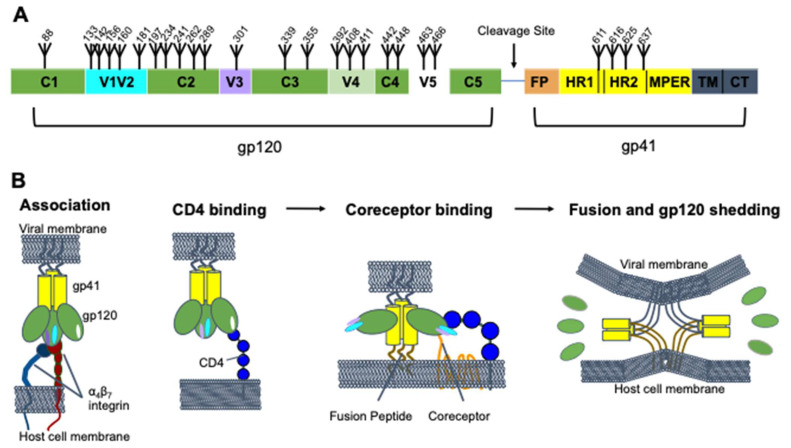
HIV-1 Env structure and dynamics. (**A**) Domain architecture of HIV-1 Env, with the gp120 and gp41 domains highlighted. The furin cleavage site, fusion peptide (FP), heptad repeats 1 and 2 (HR1 and HR2), membrane proximal external region (MPER), transmembrane domain (TM), and cytoplasmic tail (CT), are shown. Glycosylation sites are numbered (based on the HXB2 strain) and marked with fork symbols. (**B**) Viral entry. The gp41 (yellow) and gp120 (green) subunits are shown with their variable loops, V1V2 (cyan), V3 (purple), and V5 (white) in different conformations as they go through the fusion process. The fusion peptide (brown) and transmembrane domain (gray) are also shown. V4 is not shown in panel B, and the loops were excluded from the fusion panel for clarity. Binding partners on the host are shown, α_4_β_7_ (red, navy), CD4 (blue), and the coreceptor (orange).

HIV-1 Env is a heavily glycosylated class 1 trimeric fusion protein [16,17,18]. Env is synthesized as a single gp160 molecule and is post-translationally cleaved by furin to form a gp120 and gp41 heterodimer [16,19,20]. The complex is anchored to the membrane via the transmembrane domain of gp41, which interacts with the amino terminus of the gp120 trimer to hold the two subunits together [21]. While much of the Env structure is conserved across different viral strains, there are five loops in the gp120 chain that are highly variable across different strains, called V1V2, V3, V4, and V5 (Figure 1A). The V1V2 loops protrude from the distal end of the inner domain of the gp120 trimer at the trimer apex, and the V3 and V4/V5 loops are on the distal and proximal side of the outer domain, respectively.

Env mediates viral entry by engaging first with the primary cellular receptor, CD4, and then with one of two coreceptors, either CCR5 or CXCR4 [22] (Figure 1B). gp120 binding to CD4 leads to a large conformational change in which the trimer “opens”. Specifically, this involves a rearrangement of the V1V2 and V3 loops and formation of a bridging sheet [22,23]. This conformational change then enables coreceptor binding [22,24]. Coreceptor binding leads to activation of the gp41 fusion peptide, which inserts into the target cell membrane. The gp41 protein then folds into a hairpin structure in which the two heptad repeats, HR1 and HR2, form a six-helix bundle, bringing the viral and host cell membranes into contact to complete fusion [25,26]. The α_4_β_7_ integrin has also been suggested to play an important role in HIV entry by interacting with the gp120 V2 loop of certain viral strains [27,28]. Antibodies can neutralize the virus by interfering with any of these steps.

## 3. HIV-1 Mechanisms for Evading Antibody Neutralization Responses

Due to the conformational changes Env can undergo, accessibility of epitopes to neutralizing antibodies can be limited, particularly to the CD4bs [29]. This conformational masking can also divert antibody responses and lead to non-neutralizing antibody responses, such as to nonfunctional Env proteins left on the viral surface after viral shedding [30].

In addition to evading immune responses through structural heterogeneity, HIV-1 Env also effectively evades antibody recognition through its high mutation rate. The lack of proofreading mechanisms by its reverse transcriptase allows HIV-1 Env to evolve quickly in response to selective pressure from the host immune response [31,32]. The rapid mutation rate of HIV-1 differentiates it from other viruses in that it generates incredible genetic diversity within a single host throughout the course of infection, in addition to its diversity across different populations worldwide [33,34].

**Figure 2:**
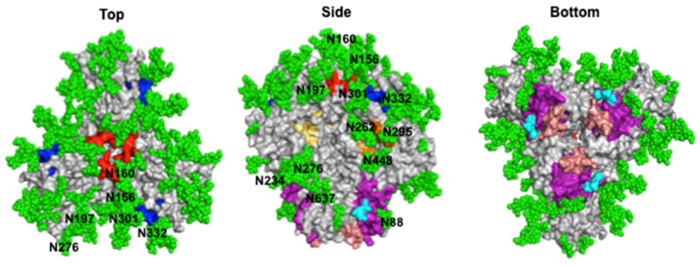
HIV-1 Env glycan shield and broadly neutralizing epitopes. Surface representation of the fully glycosylated BG505 HIV-1 Env (PDB ID: 5T3Z) is shown from different views along with glycans (green spheres labeled with the amino acid that is glycosylated) and broadly neutralizing antibody epitopes colored based on site: CD4bs (yellow), V1V2 (red), V3 glycan supersite (blue), silent face (orange), gp120–gp41 interface (purple), and the gp41 FP (cyan) and a portion of the MPER (salmon).

Env also has a “glycan shield” that sterically blocks antibodies from accessing much of its protein surface, with each Env containing 26–30 high-mannose or complex N-linked glycans [17,35] (Figure 2). As viral glycans often resemble “self” within the host, antibodies are less likely to recognize them without peptide sequences, or else they may have some level of autoreactivity [36]. Additionally, mutations in Env can impact glycosylation patterns on its surface, increasing its variability and impacting the neutralization potential of antibodies [37].

## 4. HIV-1 Neutralizing Epitopes

Despite HIV-1 defense mechanisms, bnAbs targeting a significant number of viral variants have been produced by some HIV-1 infected individuals, as well as by mice or other animals. Their identification can be partly attributed to the use of disulfide-stabilized constructs of Env, called “SOSIPs”, which mimic the native ectodomain of the trimer, as a probe [38,39]. SOSIPs have also contributed to antibody–Env complex structure determinations and neutralizing epitope characterizations. To date, bnAbs have been shown to target six epitopes on HIV-1 Env: the CD4bs, the V1V2 loops at the trimer apex, the V3 glycan supersite, the gp120–gp41 bridging region, the silent face, and gp41 (Figure 2). Recently identified bnAbs are listed in Table 1 and their structural features are described below.

### 4.1. CD4 Binding Site

The CD4bs is of particular interest because antibodies against this site can prevent interaction with CD4, the primary receptor for viral entry. The CD4bs lies in a cavity formed at the interface of the gp120 inner and outer domains (Figure 2) [22]. Hydrophobic residues deep in the pocket allow for contacts with Phe-43 of CD4 [51]. As a conserved immune target, the epitope is gated by the V1, V2, and V3 loops, as well as multiple N-linked glycans, notably at positions N197, N262, and N276 [52]. Classically, two overarching classes of CD4bs-directed bnAbs have been identified: the first class are CD4 mimics, which contact the CD4bs with heavy chains encoded by either V_H_1-2 or V_H_1-46, while the second class are “CDRH3-dependent bnAbs”, since their interactions with the CD4bs are dominated by the CDRH3 loop [53].

Recent advances have identified five CD4-mimic bnAbs [40,43] of which three fall within the VRC01 subclass, characterized by a V_H_1-2-derived heavy chain and a short, 5-residue CDRL3 used to avoid clashes with the N276 glycan [40,43]. PGV19 is a VRC01-class bnAb that neutralized 70–75% of a 20-pseudovirus panel, and is the first structurally characterized VRC01 class bnAb with a lambda light chain [40,54]. Nonetheless, only subtle differences were found between the binding modes of PGV19 and previously characterized VRC01 class bnAbs. Two other VRC01 subclass bnAbs, 2411a and 2413a, have been identified from humanized mice and are discussed in Section 5.

**Figure 3:**
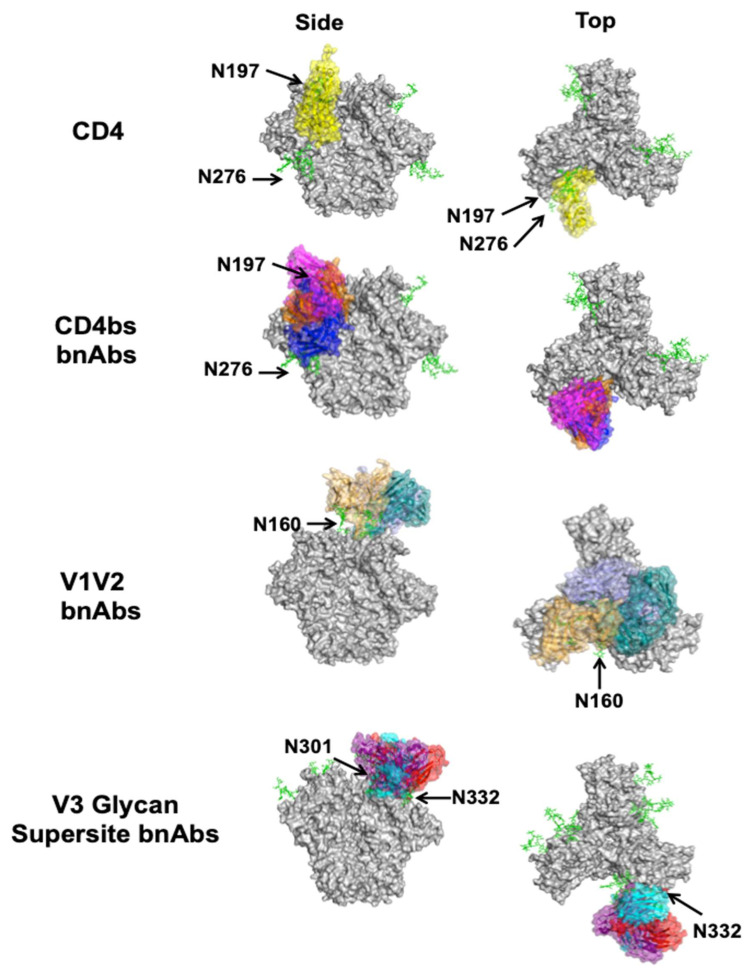

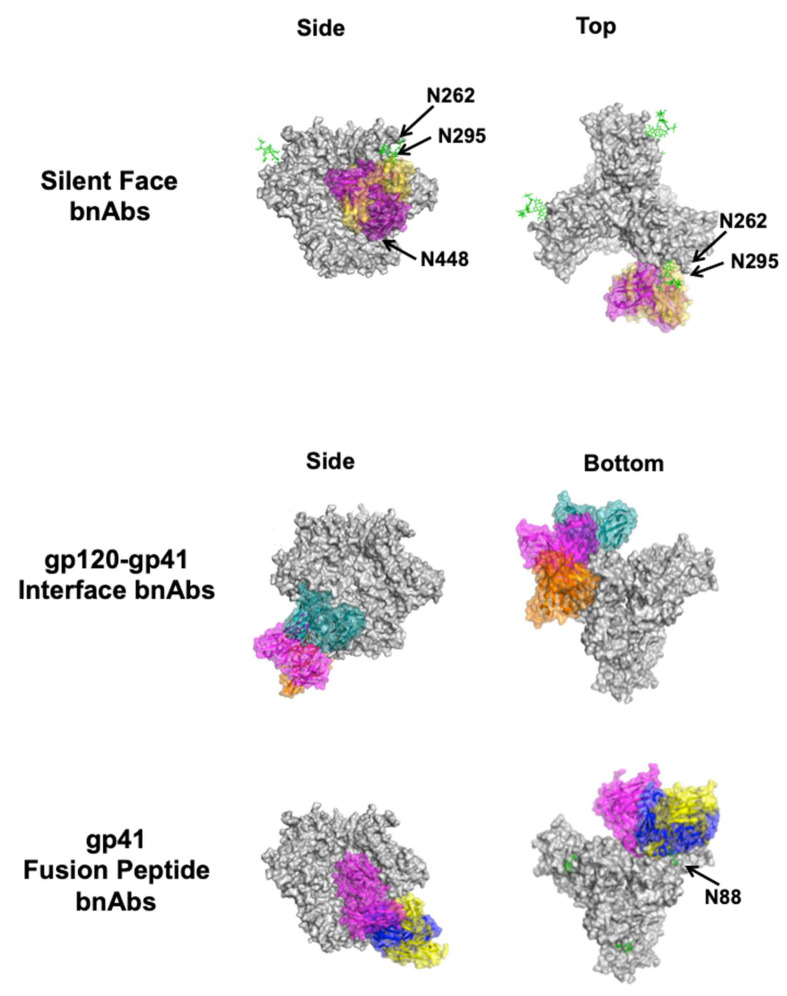
Complexes between bnAbs and HIV-1 Env. Top and side views of CD4 (yellow, PDB ID: 1GC1) in complex with the Env trimer (gray, PDB ID: 5T3Z) are shown for reference along with CD4bs bnAbs VRC01 (blue, PDB ID: 4LST), CH103 (orange, PDB ID: 4JAN), and M1214-N1 (magenta, PDB ID: 6VY2) below. Of the V1V2 apex bnAbs structurally characterized to date, CAP256-VRC26.25 (teal, PDB ID: 6VTT), VRC38 (purple, PDB ID: 5VGJ), and PGT145 (orange, PDB ID: 5V8L) are shown. Of the V3 glycan supersite bnAbs structurally characterized to date, DH270.6 (cyan, PDB ID: 6UM6), 438-B11 (red, PDB ID: 6UTK), and Ab1485 (purple, 7KDE) are shown. The two known silent face bnAbs SF12 (magenta, PDB ID: 6OKP) and VRC-PG05 (yellow, PDB ID: 6BF4) are shown. Of the gp120–gp41 interface bnAbs structurally characterized to date, 8ANC195 (teal, PDB ID: 5CJX), 35O22 (magenta, PDB ID: 4TVP), and 1C2 (orange, PDB ID: 6P65) are shown. Only gp41 FP-directed bnAbs VRC34 (blue, PDB ID: 6NC3), ASC202 (yellow, PDB ID: 6NC2), and PGT151 (magenta, PDB ID: 6DCQ) are shown; MPER-directed bnAbs clash with a closed trimer and are therefore not shown. Relevant glycans are shown as green sticks and labeled according to the amino acid that is glycosylated. For clarity, only the Fv region of the antibodies is shown on a single protomer of the trimer, even if additional copies can bind. Side (left) and bottom or top (right) views are shown.

VRC01 subclass bnAbs are generally broader and more potent than CD4-mimic bnAbs that utilize V_H_1-46 to contact the CD4bs. However, Schommers and colleagues isolated two V_H_1-46 CD4-mimic bnAbs, called 1–18 and 1–55, with superb neutralization potency, and 1–18 was found to rival or exceed the potency of all previously described CD4bs-directed bnAbs [41]. Like other V_H_1-46-encoded CD4 mimics, 1–18 and 1–55 contact the CD4bs loop, the V5 and D loops, and the N197 and N276 glycans on Env (Figure 3). They also overlap with the variable fragment of PGV19 and approach Env from a similar angle. Uniquely, however, 1–18 mimics Phe-43 CD4 contacts via residue F54_HC_ and a V5 loop salt bridge via residue R64_HC_. 1–18 also contains a six-residue insertion in CDRH1, creating a highly anionic ^25^DDDPYTDDD^33^ motif. This motif is more ionic and makes more interactions with a conserved cationic patch on the neighboring gp120 protomer than has been previously described for other bnAbs of this class. In accordance with the potency of 1–18, it was found to suppress viral loads in humanized mouse models more effectively than other CD4bs-targeting bnAbs, and it was resistant to VRC01 class escape mutations, indicating promise in potential combination therapies.

Unlike CD4-mimic bnAbs, CDRH3-dependent bnAbs have a different approach angle on Env. Jia and colleagues identified M1214_N1, which extends its long CDRH3 into a groove formed by the N197 and N386 glycans to contact the integrin binding site between the V1V2 C and C’ strands [55]. In doing so, M1214_N1 approaches the CD4bs closer to the trimer apex than CD4 mimics, similar to CH103 [56] (Figure 3). The epitope is centered on the CD4 binding loop and extends from V2 to V5, dubbed the “V2V5 corridor”. The conservation of this epitope resulted in 65% breadth, but the lower conservation in V2 contacts make M1214_N1 less broad than other CD4bs bnAbs [42]. Additionally, like other CD4bs-directed bnAbs, M1214_N1 had high levels of somatic hypermutation (SHM)—35% SHM of V_H_3-66 and 24% SHM of V_L_2-11—which may limit its potential for elicitation following immunization. Further studies are required to validate the utility of targeting this neutralizing epitope in other donors.

### 4.2. V1V2

The V1 and V2 loops are located at the distal end of each of the three gp120 molecules (Figure 2). They form beta strands, stabilized by interstrand disulfide bridges, at the cationic Env trimer apex [55,57,58]. Surrounding these loops is a conserved region covered by glycans, most notably, the N160 glycan. The V1V2 loops undergo large conformational changes after CD4 engagement to expose the otherwise shielded V3 loop and the coreceptor binding site. Even though the V1V2 loops are the most variable on Env and are shielded by glycans, a significant number of bnAbs against this site have been isolated. These bnAbs penetrate the glycan shield via long CDRs, bind almost vertically along the three-fold axis of the trimer, and have several binding modes (Figure 3). They likely neutralize the virus by stabilizing the Env trimer and thereby interfering with structural changes needed for coreceptor binding [59].

One of the most recently structurally characterized V1V2 bnAbs in complex with HIV-1 Env is CAP256-VRC26.25 [44]. CAP256-VRC26.25 is a highly potent bnAb, more so than VRC01, and was found to neutralize 59% of viruses in a 208-strain panel. Its high potency was attributed to the ability of this bnAb to neutralize the virus in a single antibody-to-trimer stoichiometry (unlike CD4bs bnAbs), as well as its extraordinarily long CDRH3 (36 amino acids, the longest of bnAbs of this class). The cryo-electron microscopy (cryo-EM) structure of the CAP256-VRC26.25–Env complex also showed that CAP256-VRC26.25 combines features of different V1V2 bnAbs. For example, it has two sulfated tyrosines that insert into the cationic hole at the three-fold trimer axis, like PGT145 [60]. Moreover, like CH03 in the PG9 class [57], CAP256-VRC26.25 forms intermolecular beta sheet-like hydrogen bonds with the C strand of the V1V2 loop [58]. The interactions between CAP256-VRC26.25 and Env differ from VRC38, which is an outlier in this group in that it has a much shorter, nearly neutral CDRH3 and, unlike the PG9 and PG145 class bnAbs, primarily interacts with the V1V2 complex through side chain interactions [61]. While the N130, N156, and N160 glycans were observed to contact CAP256-VRC26.25, the glycan dependence of this bnAb has yet to be investigated through biochemical studies. Indeed, several bnAbs of this class have been shown to depend on glycans, though the specific glycans can vary from one antibody to the next.

### 4.3. V3 Glycan Supersite

BnAbs against the V3 glycan supersite have been isolated from numerous infected individuals [62,63,64,65]. The V3 glycan supersite is located near the V1V2 region on HIV-1 Env. The base of the V3 loop is located on the opposite side of the gp120 lobe from the CD4bs (Figure 2). It contains a highly conserved ^324^GDIR^327^ motif, and various high-mannose glycans, most notably the N332 glycan contacted by all known bnAbs targeting this site [47,48,62,63,65]. Conservation at the base of the V3 loop is likely due to its role in CCR5 coreceptor recognition [64,66], so neutralization by bnAbs targeting this epitope is likely mediated by ablating coreceptor binding, or restricting conformational changes in the V3 loop that would occur following coreceptor binding to enable gp41-mediated membrane fusion [67].

V3 glycan supersite bnAbs generally have different binding approaches on Env and are more angled relative to the three-fold axis of the trimer than V1V2-directed bnAbs (Figure 3). While many V3 glycan supersite bnAbs have been reviewed elsewhere [68,69,70], three more recent high-resolution structures of bnAbs in complex with Env have been determined, two of which are from infected human subjects. DH270.6 was first described in 2017 [62], and found to be a potent neutralizer with 55% neutralization breadth with only 12.9% SHM. Its epitope was later characterized using a glycopeptide mimic of the V3 glycan supersite and recently confirmed in complex with the Env trimer [46,71], showing contacts with both the N332 glycan and GDIR motif. While contacts with the N301 glycan were observed, they were not necessary for binding or neutralization [62]. Like some bnAbs against other epitopes, a single extended CDR loop is necessary for V3 glycan supersite bnAbs to penetrate the high-mannose patch and make contacts with the Env protein surface, but DH270.6 is able to use a shorter CDR (20 amino acids).

Antibody 438-B11 achieved greater breadth than DH270 (up to 67%), with similar neutralization potency but higher SHM (25%) [47]. Despite distinct heavy chain genes (IGHV1-2 for DH270, IGHV1-69*01 for 438-B11) and being rotated roughly 90 degrees relative to DH270.6 in its approach to Env, like DH270, 438-B11 uses its long CDRH3 loop (21 residues) to contact the GDIR motif and N332 glycan, while other parts of the heavy chain interact with the N301 glycan (Figure 3).

### 4.4. Silent Face of Gp120

The so-called “silent face” of HIV-1 Env is located on a highly glycosylated region of the gp120 outer domain across from the CD4bs, on the same side of the gp120 lobe as V3 glycan supersite but closer to the gp41 region (Figure 2) [36]. The epitope predominantly includes the N262, N295, and N448 glycans on a single gp120 monomer (Figure 3). Silent face antibodies have been suggested to neutralize HIV by inhibiting conformational changes in Env necessary for full receptor binding and cell entry [36]. The neutralization mechanism may also involve the N262 glycan, which was observed to be important for CD4-mediated viral entry [72].

SF12 and VRC-PG05 [36,49] are the only known bnAbs against the center of the silent face epitope that have been structurally characterized to date (Figure 3). VRC-PG05, previously reviewed by Sok and Burton [70], makes extensive contact with the three glycans of this epitope, in addition to some contact with gp120 peptide residues. Until the identification of VRC-PG05, 2G12 was the only known bnAb to target such a glycan-rich region [73]. In contrast to silent face bnAbs, however, 2G12 does not require any Env peptide for its interaction, and it binds closer to the CD4bs. SF12 also recognizes the three glycans of the silent face epitope, but makes more protein contacts than VRC-PG05, which might explain its greater breadth and potency. The binding approach of SF12 on Env is rotated roughly 90 degrees relative to VRC-PG05, but it still interacts with the N448 glycan in a similar way as VRC-PG05. The differences in binding modes for these two bnAbs can be explained in part by their divergent gene usages—SF12 employs V_H_4-59*01 and V_K_3-20*01, whereas VRC-PG05 employs V_H_3-7*01 and V_K_4-1*01.

### 4.5. Gp120–Gp41 Interface

Located below the gp120 lobe is the gp120–gp41 bridging region, or interface. A diverse set of bnAbs targeting this epitope have been characterized, with different binding approaches on Env and some with entirely non-overlapping epitopes and varying dependency on glycans [50,74,75,76,77]. Some bnAbs bind to the gp41 region closer to the viral membrane, whereas others bind closer to the gp120 lobe (Figure 3). Most of these bnAbs are specific for the trimer (rather than binding a single Env protomer) and are dependent on gp120–gp41 cleavage. BnAbs against this site have normal CDR lengths and neutralize Env by several mechanisms. Some bnAbs, such as 8ANC195, can prevent the conformational changes required for membrane fusion, whereas others, such as 3BC176 and 3BC315, destabilize the trimer [50,74,75,76,77]. These latter bnAbs encourage trimer dissociation by disrupting the tryptophan clasp that stabilizes the trimer base [78]. No new bnAbs from human donors against this site have been structurally characterized since the most recent reviews [14,70,79]. One recently identified bnAb against the gp120–gp41 interface, 1C2, was isolated from rabbits and is discussed in Section 5.

### 4.6. Gp41

The gp41 region of HIV-1 Env consists of multiple immunogenic domains, including the fusion peptide (FP) and membrane-proximal external region (MPER), both of which have known epitopes for bnAbs (Figure 2). Both of these domains serve important roles in host cell entry, in particular through facilitating membrane fusion after recognition of CD4 and coreceptors [80,81].

The FP is largely disordered and hydrophobic due to its interaction with the target cell membrane in both the intermediate and postfusion states [80,82,83]. In the prefusion state, the FP is sequestered in a hydrophobic region of the Env trimer [84]. BnAbs against the FP include VRC34 [85], ACS202 [74], and PGT151 [86]. Notably, in addition to interacting with the FP N-terminal residues, some bnAbs against this site also interact with complex glycans near the gp120–gp41 interface, particularly the N88 glycan. Their binding approaches on Env can vary and are similar to those of gp120–gp41 interface bnAbs (Figure 3).

The MPER is proximal to the viral membrane in the gp41 ectodomain. It is a challenging epitope for immunogen design since bnAbs against this site typically recognize both a lipid component from the viral membrane and a transient Env conformation via long CDR loops [87]. Nevertheless, numerous antibodies against this highly conserved region have been identified: DH511 [88], 4E10 [87,89], 10E8 [90], 2F5 [91], VRC42 [92], LN01 [93], PGZL1 [94], Z13E [95], and CAP206-CH12 [96], and reviewed elsewhere [70,97]. No new bnAb structures against this epitope have been reported since.

## 5. BnAb Elicitation in Mice and Other Animals

Proper non-human primate models would be helpful for testing prophylactics and therapies before using them in humans. Indeed, several groups have made progress in working with infected rhesus macaque models that have been produced using simian–human immunodeficiency virus (SHIV). Using a SHIV, Roark and colleagues isolated a V1V2 antibody called RHA1.V2.01 that had significant breadth, neutralizing 49% of viruses in a 208-strain panel [45]. Its cryo-EM structure revealed that the single RHA1.V2.01 on the trimer binds at a slightly different angle from PGT145, but is similar to PGT145 in that it has a long linear beta hairpin CDRH3 that is sulfated and reaches into the apex hole [45,60]. Additionally, bnAb Ab1485 was isolated by Wang and colleagues from a SHIV-infected macaque, and binds Env with a similar orientation to the V3 glycan supersite bnAb 438-B11 [48]. Like 438-B11, Ab1485 contacts the GDIR motif using its CDRH3 and CDRH1 loops. While Ab1485 was found to have greater potency than 438-B11 (median IC_50_ = 0.055 µg/mL), it had reduced breadth with 38.1% neutralization of a 42-pseudovirus panel. As these bnAbs resembled binding to Env of human V1V2 and V3 glycan supersite bnAbs, their elicitation in rhesus macaques highlights the utility of using these animals for studying HIV-1 bnAb development.

Elicitation of antibodies with great breadth by vaccination is a major goal of HIV-1 immunogen design. The abundance of structural data of HIV-1 Env alone and in complex with bnAbs has led to the identification of critical features important for their interactions and for immune evasion. Due to the dense glycan shield on Env, one approach researchers have used is to remove glycosylation sites from HIV-1 Env to elicit greater immune responses during initial primes. Boosting regimens would then use Envs in which glycans have been included to help focus the immune response on a desired region. This has produced some promising results. For example, Chen and colleagues isolated VRC01 class bnAbs, 2411a and 2413a, from sequential immunizations of humanized mice expressing unmutated VRC01 class precursors. These bnAbs achieved moderate breadth and potency (2411a: 51% breadth, median IC_50_ 1.49 µg/mL; 2413a: 34% breadth, median IC_50_ 3.86 µg/mL) [43]. As another example, Dubrovskaya and colleagues isolated a gp120–gp41 interface bnAb, called 1C2, from sequential immunization of rabbits [50]. This bnAb had even greater breadth than 3BC315 even though the binding footprint of 1C2 was similar to that of 3BC315. Moreover, 1C2 is similar to gp120–gp41 interface bnAbs 3BC176 and 3BC315 in its ability to destabilize Env [50,76,98]. BnAbs have also been identified in a variety of fusion peptide-vaccinated animal models [99,100], and their structures have been reviewed previously [97]. While it is promising that immunization can produce bnAbs, it remains to be seen whether these responses can be recapitulated in non-human primates and whether they can withstand viral challenge. Furthermore, the protective efficacy of bnAbs against viraemia in vivo in humans also needs to be explored.

## 6. Conclusions and Future Perspectives

Despite having identified bnAbs over 30 years ago, an HIV-1 vaccine is yet to be developed. Immunogen design has indeed improved over the past decade, largely due to advances in cryo-EM, the development of SOSIP versions of Env, and increases in the available antibody–Env complex structures [38,39]. However, improvements and/or combinations of Envs may still be necessary to mimic the complex bnAb evolution processes observed in HIV-1 infected individuals. HIV-1 mutates rapidly resulting in an “arms race” in infected subjects, whereby the immune response evolves in accordance with viral evolution, and bnAbs typically take at least 2–4 years to develop. Additionally, bnAbs have structural and genetic features that may prevent their elicitation, such as long CDR loops, restricted germline usages, and high rates of somatic hypermutations. Due to the limited number of human subjects who produce bnAbs and that have been studied longitudinally [56,62,101], it becomes imperative to produce other models in which these co-evolutionary trajectories and bnAb development can be studied. The development of SHIVs holds promise in this regard.

With the increasing number of antibody–Env complex structures available, it is expected that there will be advances in antibody therapies. Indeed, antibody combination treatments have shown promise in the clinic [102,103]. Structural information has also led to advances in antigen design, with self-assembling protein nanoparticle immunogens showing promise in directing antibody responses towards broadly reactive epitopes [104,105,106,107,108,109]. We do note, however, that the structures alone do not indicate the important contacts made between the bnAbs and Env. Neutralization assays will be needed to assess mutations at different contact points to determine the “hot spots” in each complex. This is important because HIV-1 resistance can cause treatments by antibodies to fail. Pinpointing hotpots at antibody–spike interfaces will provide insights on effective antibody combinations to help overcome viral escape mutations and perhaps provide synergistic effects. These studies will also be important for immunogen design.

## Figures and Tables

**Table 1:** HIV-1 bnAbs whose high-resolution structures have recently been determined.

Epitope	bnAb	PDB ID	Neutralization Potency (IC_50_)	Breadth (%)	Year Published	Reference
CD4bs	PGV19	6B0N	0.34–1.3 µg/mL	70–75	2018	[40]
	1–18	6UDJ	0.048 µg/mL	97	2020	[41]
	1–55	6UDK	0.096 µg/mL	92	2020	[41]
	M1214_N1	6VY2	0.19 µg/mL	65	2020	[42]
	2411a	7JKS	1.49 µg/mL	51	2021	[43]
	2413a	7JKT	3.86 µg/mL	34	2021	[43]
V1V2	CAP256-VRC26.25	6VTT	0.012 µg/mL	59	2020	[44]
	RHA1.V2.01	6XRT	0.35 µg/mL	49	2021	[45]
V3 glycan supersite	DH270.6	6UM6	0.08 µg/mL	55	2019	[46]
	438-B11	6UTK	0.18 µg/mL	67	2020	[47]
	Ab1485	7KDE	0.055 µg/mL	38	2020	[48]
Silent face	SF12	6OKP	0.20 µg/mL	62	2019	[49]
gp120–gp41 interface	1C2	6P65	8.03 µg/mL	85	2019	[50]
gp41	N/A	N/A	N/A	N/A	N/A	N/A

## Data Availability

Publicly available datasets were analyzed in this study. The data can be found in the Protein Data Bank (PDB) archives using PDB IDs displayed in Table 1.
